# Dataset on transcriptional profiles and the developmental characteristics of PDGFRα expressing lung fibroblasts^☆^

**DOI:** 10.1016/j.dib.2017.06.001

**Published:** 2017-06-07

**Authors:** Mehari Endale, Shawn Ahlfeld, Erik Bao, Xiaoting Chen, Jenna Green, Zach Bess, Matthew Weirauch, Yan Xu, Anne Karina Perl

**Affiliations:** a Perinatal Institute, Division of Pulmonary Biology, Cincinnati Children׳s Hospital Medical Center, Cincinnati, OH 5229-3039, United States; b Center of Autoimmune Genomics and Ethology, United States

**Keywords:** Lung, PDGFRα-fibroblast, Transcription, Development

## Abstract

The following data are derived from key stages of acinar lung development and define the developmental role of lung interstitial fibroblasts expressing platelet-derived growth factor alpha (PDGFRα). This dataset is related to the research article entitled “Temporal, spatial, and phenotypical changes of PDGFRα expressing fibroblasts during late lung development” (Endale et al., 2017) [1]. At E16.5 (canalicular), E18.5 (saccular), P7 (early alveolar) and P28 (late alveolar), PDGFRα^GFP^ mice, in conjunction with immunohistochemical markers, were utilized to define the spatiotemporal relationship of PDGFRα^+^ fibroblasts to endothelial, stromal and epithelial cells in both the proximal and distal acinar lung. Complimentary analysis with flow cytometry was employed to determine changes in cellular proliferation, define lipofibroblast and myofibroblast populations via the presence of intracellular lipid or alpha smooth muscle actin (αSMA), and evaluate the expression of CD34, CD29, and Sca-1. Finally, PDGFRα^+^ cells isolated at each stage of acinar lung development were subjected to RNA-Seq analysis, data was subjected to Bayesian timeline analysis and transcriptional factor promoter enrichment analysis.

## Highlights

• Spatiotemporal and transcriptional analysis of PDGFRα-expressing fibroblasts during acinar lung development.
• The RNA-Seq data provide a vast source to study PDGFRα^+^ fibroblasts in lung development and disease.
• The transcription factor promoter enrichment analysis identify specific targets of PDGFRα^+^ fibroblast function.

**Specifications Table**

**Table:** 

Subject area	Developmental Biology
More specific subject area	Lung Development
Type of data	Table, image, text file, graph, figure
How data was acquired	3D Confocal Microscope inverted A1Rsi (Nikon Instruments, Melville, NY), fluorescent activated sorting flow cytometry (LSR II, BD Bioscience), MACS microbeads (Miltenyi Biotec technology, Gladbach, Germany), RNA-Seq (Illumina Inc. San Diego, CA, USA).
Data format	Filtered, analyzed
Experimental factors	Samples were not pretreated
Experimental features	The transcriptional profile, temporal, spatial and functional roles of PDGFα^GFP^ expressing fibroblasts were examined at different stages of acinar lung development using RNA-Seq, confocal microscopy and flow cytometry, respectively.
Data source location	Cincinnati, OH 45229, USA
Data accessibility	Data is incorporated with this article
	Data is accessible at: https://research.cchmc.org/pbge/lunggens/mainportal.html

## 1 Data

**Fig. 1:**
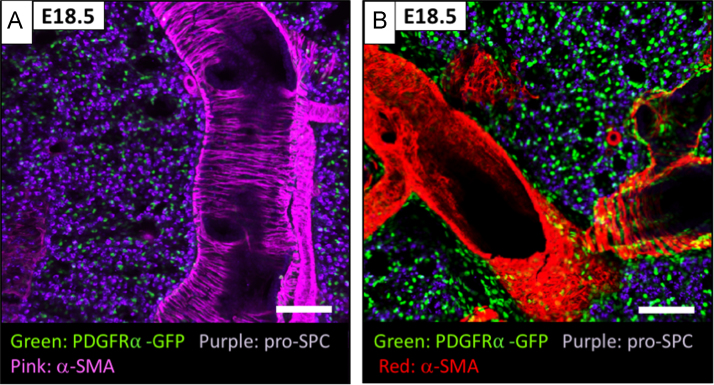
Spatial distribution of PDGFRα^GFP^ cells during the saccular stage of development. Confocal microscopy of lung sections from E18.5 PDGFRα^GFP^ mouse lungs co-stained with αSMA and pro-SPC to demonstrate the relationship of PDGFRα^GFP^ cells to saccular epithelial cells and contribution to αSMA-containing developing conducting airways (A) and blood vessels (B). Images obtained with 40X objective.

**Fig. 2:**
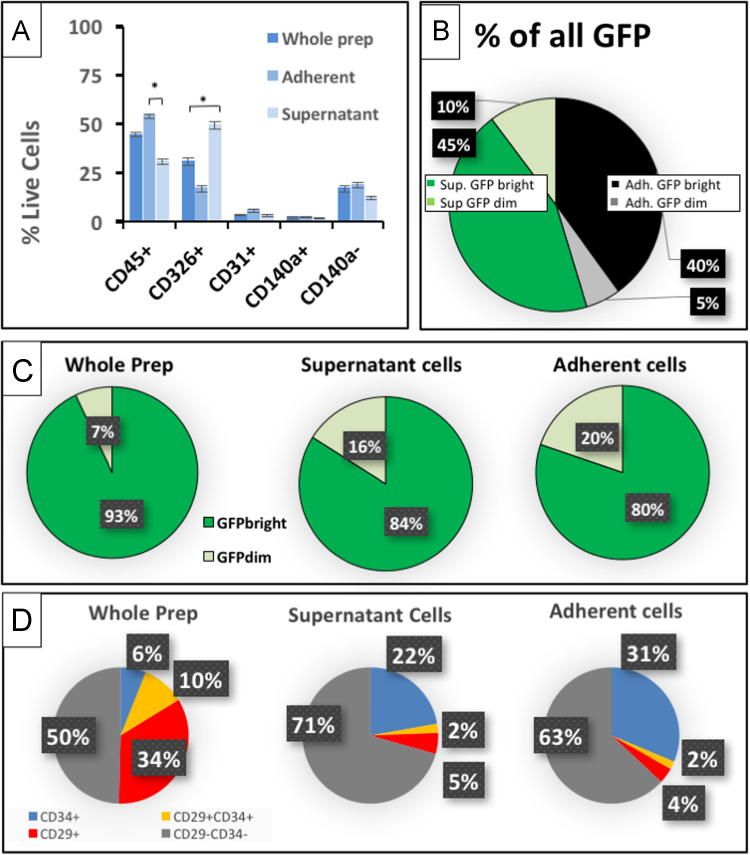
Proportions and characteristics of PDGFRα^GFP^ cells obtained by direct FACS compared to isolation by differential adherence. Flow cytometry profile of lung cell lineage proportions and PDGFRα^GFP^ proportions of fresh whole cell suspension or after selection by differential adherence. Single cell suspensions were stained and subjected to FACS directly after isolation (whole prep) or following incubation for 2 h to obtain non-adherent cells (supernatant) and adherent stromal cells (adherent). (A) Relative proportion of hematopoietic (CD45^+^), epithelial (CD326^+^), endothelial (CD31^+^), CD140a^+^ (PDGFRα^+^) stromal, and CD140a^neg^ stromal cell populations analyzed by FACS in samples obtained from whole-lung suspension vs. non-adherent and adherent cells following differential adherence. (B) Distribution of PDGFRα-GFP^bright^ and PDGFRα-GFP^dim^ stromal cells contained in non-adherent and adherent cell fractions following differential adherence. (C) Proportion of PDGFRα-GFP^bright^ (dark green) and PDGFRα-GFP^dim^ (light green) fibroblasts contained in single cell suspensions by direct FACS or FACS following differential adherence. (D) The relative proportion CD29^+^, CD34^+^CD29^+^, CD34^+^ or CD29^-^CD34^-^ subpopulations in CD140a^+^ cells analyzed by direct FACS or FACS following differential adherence.

**Fig. 3:**
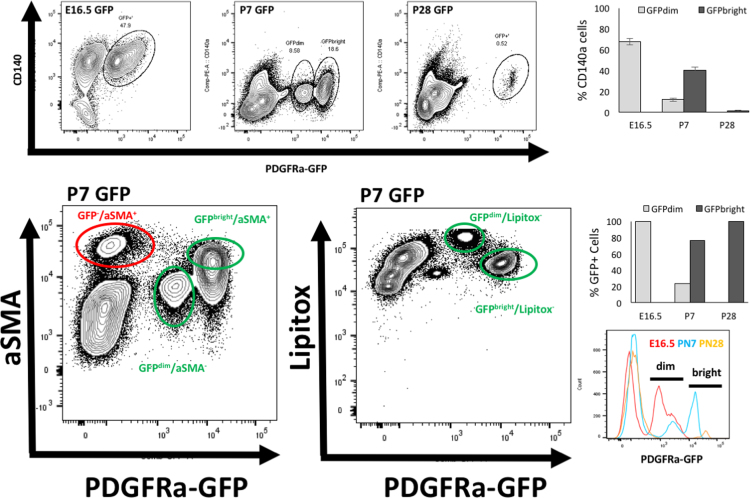
Assessment of PDGFRα-GFP interstitial fibroblasts for PDGFRα-GFP^dim^ and PDGFRα-GFP^bright^ subpopulations. Flow cytometry profiles of PDGFRα^GFP^ dim and bright sub-populations throughout acinar lung development. (A) Relative proportions at E16.5, P7, and P28 of PDGFRα-GFP^dim^ and PDGFRα-GFP^bright^ subpopulations. (B-C) Contribution of αSMA^+^ myofibroblasts (B) and LipidTOX^+^ lipofibroblasts (C) to the discernible PDGFRα-GFP^dim^ and PDGFRα-GFP^bright^ subpopulations that comprise the pool of P7 PDGFRα-GFP interstitial fibroblasts. (E) Quantification of GFP^dim^ and GFP^bright^ in CD140 positive cells. (F) Quantification of GFP^dim^ and GFP^bright^ in all GFP positive cells. (F) Histogram overlay of GFP intensity in E16.5, PN7 and PN28 GFP cells.

**Fig. 4:**
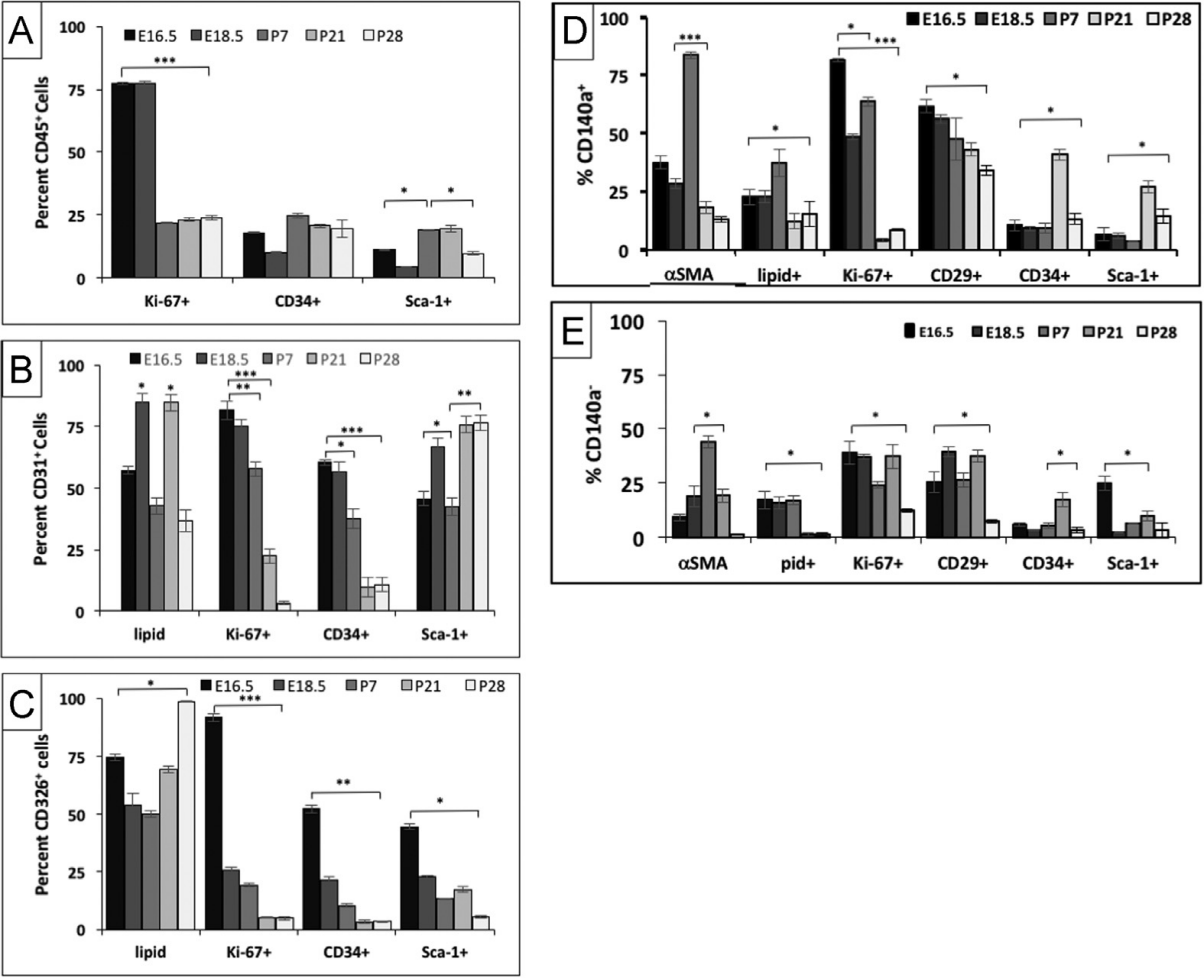
Temporal profiles of proliferation, neutral lipid, αSMA and surface marker expressions of CD45^+^, CD326^+^, CD31^+^, CD140a^+^ stromal and CD140a^neg^ stromal cell lineages. Proliferation, αSMA, lipid, CD29, CD34 and Sca-1 expressions of CD45^+^ hematopoietic (A), CD31^+^ endothelial (B), CD326^+^ epithelial (C), CD140a^+^ stromal (D) or CD140a^-^ stromal (E) cells at E16.5 (canalicular), E18.5 (saccular), P7 (early alveolar), P21 (mid alveolar), and P28 (late alveolar) stages of acinar lung development. Data is presented as the relative percentage of cells within each individual cell lineage.

**Fig. 5:**
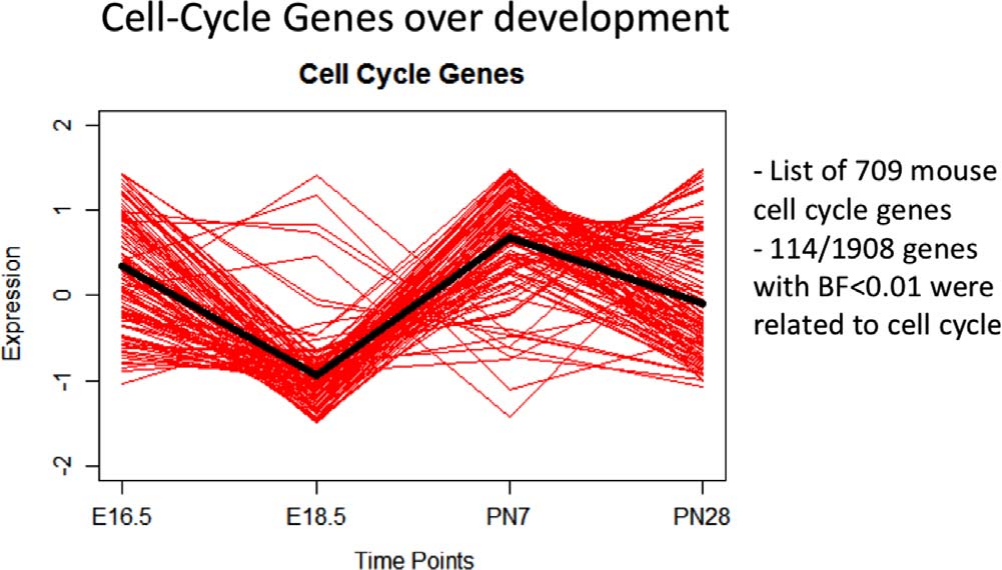
Transcriptional profile of cell cycle genes expressed in PDGFRα^+^ fibroblasts throughout critical stages of acinar lung development. Expression profile obtained by Bayesian and STEM analysis of RNA-Seq data to identify cell cycle genes that are differentially expressed in PDGFRα^+^ fibroblasts between E16.5, E18.5, P7, and P28 during acinar lung development.

The data presented herein are representative of the key stages of acinar lung development and define the developmental role of lung interstitial fibroblasts expressing platelet-derived growth factor alpha (PDGFRα). Cells expressing PDGFRα were analyzed at E16.5, E18.5, P7 and P28. The spatiotemporal localization of PDGFRα^GFP^ E18.5 at (Fig. 1) demonstrates the relationship of PDGFRα^+^ fibroblasts to proximal and distal saccular lung structures. Flow cytometry using direct flow cytometry of whole-lung single cell suspension preparation and selection by differential adherence in tissue culture to enrich and analyze PDGFRα^+^ fibroblast populations is presented in Fig. 2. PDGFRα^GFP^ expression was assessed at E16.5, P7, and P28 for GFP^dim^ and GFP^bright^ sub-populations. For the two distinct sub-populations present at P7, the relative abundance of myofibroblasts (αSMA^+^) and lipofibroblasts (LipidTOX^+^) within each population is presented (Fig. 3). Fig. 4 shows data on temporal changes in neutral lipid, αSMA, proliferation, and cell surface expression of CD34, CD29, and Sca-1 in CD326^+^, CD31^+^, CD140^+^ and CD140a^neg^ stromal cells. The gene expression profile from RNA-Seq data provides information in cell-cycle gene changes of isolated PDGFRα^+^ fibroblasts throughout acinar lung development (Fig. 5 and Table 1), individual genes upregulated at E18.5 in PDGFRα^+^ fibroblasts (Table 2), and changes in contractile gene expression in PDGFRα^+^ fibroblasts (Table 3). Additionally, data from computational transcription factor binding site analyses (Table 4), ChIP-Seq enrichment profiles (Table 5), and promoter sequences of individual genes dynamically expressed by PDGFRα^+^ fibroblasts during acinar lung development. The three transcription factors identified by ChIP-Seq analysis are presented in Table 6.

**Table 1:** Cell cycle genes that are differentially expressed in PDGFRα^+^ fibroblasts during distinct stages of acinar lung development.

Gene	E16	E18	PN7	PN28
Calm2	−0.00571762	−0.870833	1.40198	−0.525428
Dusp1	−0.621371	−0.627324	−0.2244	1.47309
Pmp22	−0.742166	−0.979494	0.798682	0.922978
Sptbn1	−0.789379	−0.936042	0.940708	0.784713
Srsf5	0.236167	−1.08452	1.26328	−0.414932
Hsp90aa1	1.41313	−0.874767	−0.484744	−0.0536168
Rhoa	−0.135108	−1.06733	1.34942	−0.146979
Tubb5	0.832279	−0.958058	0.894011	−0.768232
Thbs1	0.872403	0.828917	−0.622006	−1.07931
Gnai2	−0.147148	−1.03287	1.36953	−0.18952
Jun	−0.851524	−0.510853	−0.055664	1.41804
Anapc13	1.1925	−0.897243	−0.751497	0.456244
Gnb2l1	1.40582	−0.040552	−0.47046	−0.89481
Stmn1	0.934956	−1.09228	0.757047	−0.599728
Smc1a	0.883569	−1.00734	0.835485	−0.711719
Tuba1a	−0.354266	−0.839746	1.45038	−0.256367
Pcna	1.26778	−0.642412	0.313621	−0.938985
Rhob	−0.535957	−1.11043	0.573782	1.0726
Ywhah	0.729312	−1.24987	0.880943	−0.360389
Calm1	0.056566	−1.42903	0.766416	0.606044
Mapre2	−0.362037	−0.861711	1.44181	−0.218067
Sept2	−0.191729	−0.869314	1.43734	−0.376292
Plk2	−0.294531	−0.463503	1.47699	−0.718961
Rad21	0.830549	−1.05783	0.876974	−0.649691
Txnip	−1.04578	−0.321895	0.0252466	1.34242
Cdk4	1.21118	−0.889579	0.426562	−0.74816
Trp53	0.304485	−1.02912	1.25272	−0.528087
Ube2c	1.31111	−0.750289	0.249112	−0.809935
Sept4	−0.884936	−0.73429	0.381385	1.23784
Mapk3	−0.467964	−1.05953	1.25962	0.267868
Chd4	0.965382	−1.06658	0.73079	−0.629589
Csnk1a1	0.145345	−0.458597	1.32271	−1.00946
Sept11	0.38547	−0.834781	1.23797	−0.788659
Cetn3	0.920627	−1.39894	0.440421	0.0378935
Nsmce2	0.927752	−1.12649	0.751502	−0.552767
Ppp1cc	0.757517	−0.961919	0.962431	−0.758029
Ppp1ca	0.769955	−1.19987	0.876423	−0.446511
Smc4	1.16898	−0.798464	0.49566	−0.866176
Ppp1cb	−0.197787	−1.11343	1.31193	−0.0007138
Smc3	1.1891	−1.03988	0.422153	−0.571375
Cks1b	1.34665	−0.629637	0.15774	−0.874757
Sept7	−0.183509	−0.878618	1.43392	−0.371793
Mki67	1.18879	−0.728754	0.459143	−0.919178
Spin1	0.10549	−0.943386	1.35379	−0.515895
Top2a	1.20696	−0.806017	0.43718	−0.838125
Tubb4b	0.286298	−1.44805	0.310388	0.851369
Kmt2e	0.0343548	−1.37276	0.999874	0.33853
Rgs2	−0.0296069	1.39706	−0.446672	−0.920779
Son	0.19415	−1.31686	1.10814	0.0145653
Gnai3	0.490256	−1.24935	1.05433	−0.295233
Cdkn1c	1.41418	−0.105178	−0.397307	−0.911693
Usp9x	0.0760878	−1.4367	0.72769	0.632917
Zak	−0.700217	−1.01532	0.918107	0.797432
Ier3	0.980432	0.73242	−0.723419	−0.989433
Wapal	0.632021	−1.23307	0.971144	−0.370096
Anapc5	0.682133	−1.37308	0.798597	−0.10765
Ppm1g	0.965661	−1.34965	0.485437	−0.101447
Specc1l	−0.56199	−0.715791	−0.184349	1.46213
Nedd9	−0.579639	−1.04244	1.17282	0.449265
Ube2i	0.696443	−1.44155	0.654793	0.0903107
Sirt2	−0.128286	−1.19834	1.24105	0.0855696
Usp47	0.972221	−1.25556	0.60976	−0.326424
Nasp	1.35164	−0.650695	0.151524	−0.85247
Ccar1	1.19936	−0.85878	0.448599	−0.789176
Lats2	−0.50131	−1.12181	1.10564	0.517486
Ccny	0.0769666	−1.09766	1.30669	−0.285998
Calm3	0.207701	−1.12116	1.25756	−0.344101
Eid1	−0.207967	−0.932492	1.41784	−0.277379
Foxn3	−0.353993	−1.18568	1.14637	0.393299
Mtus1	−0.231669	−1.04971	1.35605	−0.0746687
Usp8	0.0653615	−1.32136	0.147907	1.10809
Ran	1.27067	−0.982225	0.291102	−0.579552
Cdc123	0.684283	−1.30701	0.872431	−0.249708
Ccnt1	0.350793	−1.48693	0.669501	0.466636
Ppp6c	−0.0845592	−1.25198	1.18414	0.152397
Dynlt3	−0.662532	−1.02528	1.05067	0.637141
Smarca4	0.897716	−0.966186	0.827408	−0.758938
Pafah1b1	−0.0028538	−1.05804	1.34217	−0.281278
Trp53bp2	0.0115787	−1.34168	1.06203	0.268075
Marveld1	−0.809342	−0.848667	1.18272	0.475286
Ccndbp1	−0.23958	−1.27197	1.0577	0.453849
Ccpg1	−0.647646	−0.929798	0.302994	1.27445
Cdk6	0.385309	−0.958141	1.22701	−0.654178
Ctgf	−0.476914	−1.05691	0.277427	1.2564
Khdrbs1	0.834267	−0.819132	0.89601	−0.911146
Arl8b	0.352736	−1.47068	0.34949	0.768457
Nudc	1.41244	−0.834935	−0.023113	−0.554394
Anxa1	−0.049034	−0.778171	−0.598799	1.426
Nipbl	0.583539	−1.21221	1.01876	−0.390092
Stag1	0.412112	−1.08482	1.18106	−0.508359
Junb	0.410953	1.17702	−1.09807	−0.489902
Cdk11b	0.0832837	−1.20526	1.2359	−0.113924
Cast	−0.2003	−1.33032	0.668522	0.862097
Klhl9	0.406837	−1.35494	0.998055	−0.0499484
Ctcf	0.823152	−1.16313	0.845177	−0.505199
Usp16	0.280098	−1.43447	0.891483	0.262886
Pcnp	0.473111	−1.32507	0.999318	−0.147358
Brd7	0.444032	−1.49762	0.47713	0.576461
Cdkn1a	0.0916268	0.462374	−1.4225	0.868503
Cdc5l	1.11105	−1.1579	0.483718	−0.436864
Psme3	1.21813	−1.16459	0.254701	−0.30825
Ckap5	0.976807	−0.964173	0.740723	−0.753357
Setd8	0.947553	−1.26396	0.634908	−0.318502
Erh	1.03215	−1.0091	0.665275	−0.688322
Sept9	0.555875	−0.684408	1.11649	−0.987952
Gas6	−0.610436	−0.642252	−0.219312	1.472
Yeats4	1.06306	−1.19386	0.532339	−0.401537
Ralb	0.445575	−0.909659	1.19804	−0.733953
Hcfc1	1.0087	−1.15647	0.636994	−0.489225
H2afx	1.36257	−0.760365	0.137039	−0.73924
Ccnd2	−0.26808	−0.727408	1.47332	−0.477836
Mapre1	0.403968	−0.845579	1.22686	−0.78525
Ep300	0.310167	−1.46051	0.341653	0.80869
Tacc1	−0.582128	−0.859333	0.0559869	1.38547

**Table 2:** Genes upregulated in PDGFRα^+^ fibroblasts at E18.5 relative to other stages of acinar lung development.

Gene	E16	E18	PN7	PN28
ADAMTS1	−1.0996	1.89932	−0.778615	0.109199
HBA-A1	−0.72184	1.89803	−0.602954	−0.736058
HBB-BT	−0.698284	1.89715	−0.648254	−0.725893
OLFR62	−0.658434	1.89326	−0.719626	−0.719626
HBB-B1	−0.701092	1.8915	−0.598885	−0.7459
ENPP2	−0.696722	1.89078	−0.611701	−0.668638
GM17644	−0.743782	1.88918	−0.860528	−0.196981
4930470H14RIK	−1.12671	1.88433	−0.794499	0.185705
LARS2	−0.712029	1.88186	−0.874364	−0.206367
PENK	−0.697763	1.8681	−0.466888	−0.81606
PROS1	−1.42972	1.85969	−0.092791	−0.456466
MT1	−0.547424	1.82338	−0.993104	−0.15854
GM10052	−0.166882	1.70856	−0.893127	−0.893127
TGFBR3	−1.64478	1.69903	−0.353873	0.405727
HBB-Y	−0.127185	1.68753	−0.892553	−0.910755
TRIB1	−0.115112	1.65209	−1.06878	−0.363624
RGS2	−0.070595	1.59547	−0.557646	−1.11131
ALDH2	−0.916657	1.55663	−1.19667	0.944171
NDRG2	−0.78057	1.55655	−1.26841	0.882676
ODC1	−0.038573	1.55499	−0.483119	−1.21095
HHIP	0.188624	1.48161	−0.840506	−0.864842
SPRED1	−0.149031	1.45532	−0.070748	−1.56369
ZFP36	0.226683	1.45129	−1.11433	−0.463834
BC117090	0.303844	1.42934	−1.00008	−1.00008
HBA-X	0.373835	1.37937	−1.01109	−1.01109
MYC	0.149769	1.35186	−0.277893	−1.49285
SNORA52	0.415108	1.34884	−1.01697	−1.01697
RMRP	0.351773	1.29452	−1.3491	−0.068137
MAFF	0.437803	1.28682	−0.755838	−1.05255
JUNB	0.463468	1.27891	−1.14282	−0.495453
IIGP1	0.246262	1.27624	−0.268592	−1.53017
CCDC3	−1.24372	1.25611	0.861579	−1.34577
1500012F01RIK	0.45529	1.25174	−0.659759	−1.17327
BHLHE40	0.544517	1.19043	−1.25119	−0.321188
FAU	0.622948	1.15584	−0.922513	−0.866115
BTG2	0.632911	1.11406	−1.25966	−0.320921
HSPB1	−0.866861	1.1016	−1.24201	1.53001
RPPH1	−0.046067	1.05871	−1.60545	1.10958
KLF9	−1.24071	1.04622	−0.906275	1.56238
ATF3	0.811023	0.98054	−1.12267	−0.576751
SOCS3	0.870832	0.928448	−1.02609	−0.729075
ITGAV	−0.712633	0.916735	1.02392	−1.81532
SLC2A1	0.891161	0.905855	−1.0727	−0.655793
THBS1	0.900603	0.854342	−0.689139	−1.17562
IFRD1	0.217954	0.829998	−1.65768	1.15147
SKIL	0.157235	0.777133	0.584415	−2.05964
EIF3E	0.93836	0.777077	−0.563455	−1.31203
H19	1.03227	0.774112	−0.959012	−1.08616
CCNL1	1.02674	0.764284	−0.973248	−0.792439
IER3	1.01516	0.75419	−0.777739	−1.05766
EEF1A1	0.893956	0.731323	−0.340296	−1.53243
CDKN1A	0.256919	0.726925	−1.66259	1.24179
IGF2	1.05441	0.725075	−0.829588	−1.09723
ELN	−1.81889	0.705691	0.891242	0.0251241
LOX	1.10847	0.663704	−1.10496	−0.568685
SNORA15	1.17483	0.621342	−1.02944	−1.02944
MMP2	−0.11503	0.562767	−1.52805	1.71138
FMO2	−1.01789	0.561459	−0.900695	1.89521
PTN	1.19277	0.559803	−0.808516	−1.06382
HES1	1.13426	0.553428	−0.544956	−1.29785
TREM3	1.23746	0.5435	−1.02013	−1.02013
PLAGL1	1.11539	0.540813	−0.457605	−1.38903
SNORA75	1.24616	0.532386	−1.01866	−1.01866
GLUL	−1.67974	0.52869	0.0790104	1.29294
EDNRB	1.22549	0.501732	−0.735441	−1.08308

**Table 3:** Transcriptional profile of contractile genes differentially expressed in PDGFRα^+^ fibroblasts over the course of acinar lung development.

Gene	E16	E18	PN7	PN28
Sparc	−0.782064	−0.884034	0.502201	1.1639
Npm1	1.3844	−0.154564	−0.225484	−1.00435
Sod1	−0.106237	−1.29861	0.305218	1.09963
Vim	−0.32131	−0.867333	1.44228	−0.253635
Eln	−1.43045	0.610192	0.760175	0.0600839
Tpm1	−0.297888	−0.0921075	1.38385	−0.993852
Pfn1	1.07808	−1.01729	0.602467	−0.663255
Tmsb4x	0.783135	−1.23122	−0.396606	0.844691
Tgfbi	−0.201384	0.321878	1.13319	−1.25368
Fhl1	−0.834609	−0.825804	0.481646	1.17877
Myadm	−0.950365	−0.74732	1.06936	0.628329
Gng5	0.799333	−1.39643	0.63971	−0.0426118
Tmsb10	0.134493	−0.868777	1.35647	−0.622189
Fn1	−0.568048	−0.828443	−0.014463	1.41095
Rac1	−0.170999	−0.967267	1.402	−0.263733
Cfl1	0.952984	−0.798067	0.771927	−0.926844
Msn	−0.353353	−1.04234	1.33608	0.0596072
Arpc2	0.673331	−1.25646	0.923198	−0.340072
Fbn1	−0.745338	−0.971735	0.74445	0.972623
Itgb1	−0.573494	−0.606145	1.48646	−0.306817
Arf1	0.0803654	−1.29798	1.14149	0.0761276
Cdh11	0.801392	−1.367	0.68846	−0.122848
Rhoa	−0.135108	−1.06733	1.34942	−0.146979
Cald1	−0.306922	−0.723712	1.47733	−0.446696
Mmp2	−0.20158	0.299274	−1.24572	1.14803
Myl6	0.415826	−1.29762	1.0551	−0.173303
App	−0.830414	−0.877626	1.05587	0.652173
Ctnnb1	−0.136582	−1.31422	1.04303	0.40777
Gnai2	−0.147148	−1.03287	1.36953	−0.18952
Cdc42	0.368935	−1.11146	1.19372	−0.451196
Igf1	1.34653	0.102391	−0.472771	−0.976149
Mylk	0.0820497	−0.892443	1.37175	−0.561356
Cav1	−0.71922	−0.744583	1.38648	0.0773188
Flna	0.713322	−1.17997	0.936876	−0.470232
Gnb2l1	1.40582	−0.0405521	−0.47046	−0.89481
Tnc	−0.424942	−0.295052	1.47202	−0.752022
Tpm3	0.592418	−0.696541	1.09092	−0.986797
Capzb	0.642076	−1.23233	0.964042	−0.373784
Ctnnd1	−0.483005	−0.886022	1.4072	−0.0381741
Akap2	−0.574534	−0.49027	1.49745	−0.432647
Myh10	0.222398	−1.36676	1.03463	0.109729
Gnb1	0.0972075	−1.15717	1.26814	−0.208171
Hspb1	−0.718661	0.699476	−0.988926	1.00811
F2r	−0.749158	−0.912192	0.494055	1.1673
Ednra	1.13125	−1.15915	−0.419792	0.447694
Rhob	−0.535957	−1.11043	0.573782	1.0726
Sptan1	−0.603347	−0.903481	1.33841	0.168423
Cul3	0.560345	−1.28661	0.982377	−0.256117
Myh11	0.459331	−0.102365	0.986597	−1.34356
Pdlim3	−0.571051	0.0286009	1.39505	−0.852598
Rdx	−0.197531	−1.10317	1.32045	−0.0197473
Myh9	−0.079762	−0.635771	1.43791	−0.722382
Zyx	−0.379613	−1.08824	0.188798	1.27906
Dstn	−0.768233	−0.733085	0.140156	1.36116
Actr3	1.3969	−0.260784	−0.977504	−0.158609
Bmp4	−0.288633	−1.28364	0.914452	0.657823
Cyb5r3	−0.745596	−0.907206	1.1836	0.4692
Cdk4	1.21118	−0.889579	0.426562	−0.74816
Aldoa	0.826149	−1.14381	−0.533523	0.851188
Cdh5	−0.650899	−0.644739	1.46062	−0.164986
Ghr	0.275738	−0.76286	1.29793	−0.810804
Atp2a2	−0.51177	−1.14786	0.977006	0.682622
Ltbp2	−0.596826	−0.0823989	1.4365	−0.757278
Pls3	0.23697	−1.20358	1.20027	−0.233664
Cap1	0.554204	−1.36045	0.911563	−0.105319
Lima1	−0.323465	−1.15467	1.22366	0.254468
Dpysl2	−0.696809	−1.01872	0.807133	0.908401
Ppp1ca	0.769955	−1.19987	0.876423	−0.446511
S100a10	0.0334959	−1.01683	−0.368988	1.35232
Slc9a3r2	−0.786913	−0.833294	1.24245	0.377755
Marcks	−0.095569	−0.286677	1.38184	−0.999596
Sorbs3	−0.673773	−0.914912	1.2671	0.32158
Fus	0.644405	−0.755523	1.0575	−0.946377
Gsn	−0.512083	−0.503138	−0.484682	1.4999
Nckap1	−0.301663	−1.20117	1.16187	0.340968
Add1	−0.311211	−1.218	1.11843	0.410786
Tgfbr2	−0.717995	−0.912799	0.414436	1.21636
Cd44	0.0409719	−0.805395	1.39648	−0.632058
Tns3	−0.464562	−1.03593	0.209286	1.29121
Actn1	0.458479	−1.0762	1.15762	−0.539902
Wasf2	−0.352504	−1.07925	1.30154	0.130213
Dlc1	−0.741294	−0.879296	1.23701	0.383584
Rap1gap	−0.481962	−0.533931	−0.483672	1.49956
Dnajb6	1.06517	−1.34927	0.189553	0.0945508
Lcp1	1.41617	−0.90207	−0.101197	−0.412904
Cdkn1c	1.41418	−0.105178	−0.397307	−0.911693
Sdc4	−0.63846	−0.381346	−0.471663	1.49147
Tmod3	−0.388687	−1.17516	1.13201	0.431829
Net1	−0.145783	−0.07899	1.32632	−1.10154
Iqgap1	−0.257395	−1.23511	0.362558	1.12995
Kank2	−0.552174	−1.12744	0.921746	0.757869
Pecam1	−0.675754	−0.620807	1.45989	−0.163329
Slk	−0.082074	−0.947722	1.39944	−0.369648
Specc1l	−0.56199	−0.715791	−0.184349	1.46213
Rock2	−0.752439	−0.893999	0.449575	1.19686
Atf3	0.761761	0.925637	−1.10758	−0.579823
Actn4	−0.441928	−1.03076	1.30985	0.162832
Myo1b	−0.595032	−1.09886	0.890657	0.803232
Cxcl12	−0.377019	−0.839748	1.44875	−0.23198
Vcam1	−0.118534	−0.76449	−0.561394	1.44442
Chchd2	1.12552	−1.04178	0.525748	−0.609493
Arhgef12	−0.728362	−0.801487	1.33803	0.191823
Bcl2	0.69956	0.36696	0.417325	−1.48384
Cryab	−0.831001	−0.825524	0.470555	1.18597
Tpm2	0.358849	−0.0645877	1.0411	−1.33536
Stat3	−0.951892	−0.420543	−0.012086	1.38452
Capza1	0.945866	−0.94505	0.778122	−0.778938
Il1b	1.47332	−0.225586	−0.622641	−0.625097
Rnd3	1.34749	−0.576498	0.141145	−0.912138
Slit2	−0.72262	−0.638973	−0.075036	1.43663
Dnm2	−0.296382	−1.16068	1.23026	0.226806
Emp2	−0.964067	−0.678616	0.460195	1.18249
Marcksl1	1.2413	−0.274669	0.193641	−1.16027
Myc	0.184816	1.20839	−0.179336	−1.21387
Mif	1.49932	−0.52861	−0.458383	−0.512332
Fnbp1	0.540304	−1.46099	0.733985	0.186696
Clec2d	−0.773785	−0.863032	0.420383	1.21643
Crh	1.228	0.402804	−0.815403	−0.815403
Smarca4	0.897716	−0.966186	0.827408	−0.758938
Rhoj	−0.69625	−0.652373	1.44251	−0.0938856
Palld	−0.057783	0.00800809	1.24868	−1.19891
Pafah1b1	−0.002853	−1.05804	1.34217	−0.281278
Pik3r1	−0.845334	−0.292798	−0.311845	1.44998
Fblim1	−0.364685	−1.24792	0.737608	0.875
Cdk6	0.385309	−0.958141	1.22701	−0.654178
Ctgf	−0.476914	−1.05691	0.277427	1.2564
S1pr1	−0.673846	−0.700603	1.43239	−0.0579363
Shc1	1.07814	−0.51254	0.558318	−1.12392
Coro1b	0.085574	−1.32288	1.10712	0.130185
Mapk14	−0.241958	1.28682	−1.13058	0.0857137
Junb	0.410953	1.17702	−1.09807	−0.489902
Mprip	−0.666687	−1.02581	1.03463	0.657863
Rock1	0.158822	−1.39264	0.987159	0.246661
Hax1	0.684126	−1.47716	0.263223	0.529807
Akap13	0.0260559	−1.18589	−0.098462	1.25829
Gng12	−0.35838	−1.00589	1.36373	0.000544535
Cdkn1a	0.0916268	0.462374	−1.4225	0.868503
Gna13	−0.44997	−0.936124	1.38607	2.05e−05
Gnaq	−0.091048	−1.03193	1.36656	−0.243584
Sept9	0.555875	−0.684408	1.11649	−0.987952
Gna12	−0.300922	−0.871765	1.44129	−0.268605
Sh3pxd2a	−0.403176	−0.808365	1.45726	−0.245722
Prnp	−1.32794	0.522858	−0.162763	0.967841
Dab2	1.12043	−1.10753	−0.515332	0.502433
Mapre1	0.403968	−0.845579	1.22686	−0.78525
Fat1	0.727381	0.507463	0.234335	−1.46918
Clec7a	1.49561	−0.390523	−0.549357	−0.555734

**Table 4:** Computational transcriptional factor binding site motif enrichment analyzed in the differential gene expression pattern of six profiles.

**Profile**	**Gene/TF**	**−log *P*val**
Profile_1	KLF3	29.92
Profile_1	ELK3	29.31
Profile_1	YBX1	19.67
Profile_1	SP1	19.52
Profile_1	HBP1	12.97
Profile_1	FOXF2	6.998
Profile_1	ID3	6.854
Profile_1	CUX1	3.526
Profile_1	**CTCF**	2.103
Profile_13	KLF6	28.86
Profile_13	ELK4	13.64
Profile_13	SMARCC2	6.029
Profile_13	NFE2L1	4.113
Profile_13	NFIA	3.98
Profile_13	NFIX	3.98
Profile_13	MEF2A	3.491
Profile_13	**MAX**	2.221
Profile_13	FOXN3	3.203
Profile_10	RUNX3	4.56
Profile_10	JUNB	4.264
Profile_18	KLF7	5.862
Profile_23	FOSB	4.769
Profile_39	KLF4	36.43

**Table 5:** Previously published ChIP-Seq data with significant overlap of genes differentially expressed in CD140+ fibroblasts throughout lung development.

**Profile**	**Track**	**Cell**	**TF**	**Overlap**	**Total**	**Ratio**	**Enrichment**	***p*-Val**
Profile_1	Caltech_Tfbs	C2C12	NRSF	207	465	0.45	3.41	5.80E−66
Profile_1	Licr_Chip	MEL	CTCF	286	465	0.62	2.22	4.26E−46
Profile_13	Sydh_Tfbs	CH12	Max	193	257	0.75	2.17	1.62E−37

**Table 6:** Genes identified in the ChIP-Seq enrichment analysis and differentially expressed in CD140+ cells.

**CTCF & NRSF**	**NRSF**	**CTCF**	**MAX**
ACTN1	ANAPC5	1110004F10RIK	1700016K19RIK
ACTR2	AP2M1	1700020I14RIK	ACAA2
ANXA6	ATP5B	2700081O15RIK	ACO2
AP3B1	ATP6V0C-PS2	6820431F20RIK	ACTN4
API5	BRD2	ACAT1	ADD1
ARPC1B	CALD1	ADNP	ADIPOR1
ARPC2	CALU	ANKRD11	AHCYL1
ARPC5	CBX3	ANKRD17	AKAP12
ATP5C1	CDK11B	ANP32A	ANO6
B230219D22RIK	CFDP1	ATF7IP	AP2B1
BCLAF1	CNN3	ATP5J	ARF4
CALM2	CXCL12	ATXN2L	ARHGAP1
CALM3	DDX1	BAZ1B	ARHGDIA
CANX	DDX3X	BC005537	ARL8B
CAPRIN1	DHX15	BPTF	ASAH1
CAPZA1	DLD	BZW1	ATL3
CAPZA2	EIF5	CCND2	ATP1A1
CAPZB	FKBP10	CCNI	ATP1B3
CCNY	FOXF2	CDC123	ATP2A2
CDC42	FSTL1	CDK6	ATP6AP1
CFL1	FZD1	CDV3	ATP6V0D1
CHD4	GSK3B	CLINT1	ATP6V0E
CKAP4	GTF2A2	CNN2	ATP6V1A
COMMD3	GTF3C6	CNOT1	BAG1
COPS5	HDAC2	COPZ2	BAG6
CORO1C	HNRNPH1	CPD	BCAP31
CSNK1A1	IDH3B	CRTAP	BRD7
CUL3	IMMT	CTCF	CALM1
CYC1	ITSN2	CTDNEP1	CAPNS1
DDX39B	KTN1	CTDSP1	CAST
DENND5A	LGALS1	CUL1	CCDC47
DHX9	MDH2	CUX1	CCNDBP1
EID1	NARS	CXXC1	CCNT1
EIF3C	NCOR1	DDB1	CD164
EIF3D	NDUFA10	DNAJC10	CD47
EIF4G2	NRD1	DNAJC7	CHMP2A
EIF5B	NSMCE2	DNMT3A	CHTOP
EPB4.1L2	NXF1	DNTTIP2	CIR1
ERH	PCNP	EIF2S3X	CLIP1
EWSR1	POMP	EIF3G	CLN5
FBXO11	PPP4R2	EIF4A3	COPA
FKBP1A	PRCP	EIF4G3	COPE
FLNA	PRDX2	ELK3	COPG1
FUS	PSMB5	ERBB2IP	CR1L
GDI2	RCN2	ESF1	CRIPT
GHR	RHOA	EXT2	CSNK1G2
GLUD1	RNF4	FAM120A	CTDSP2
GNB1	RTN4	FAM193A	CTNNB1
H3F3A	SDCBP	GALNT1	CTSA
HMGB3	SETD5	GINS4	CTSB
HMOX2	SLC25A3	GNG12	CTSD
HNRNPA0	SND1	GNG5	CUTA
HNRNPA2B1	SNRNP200	GOLGA7	DAP
HNRNPDL	SNRNP70	GPBP1	DAZAP2
HPRT	STRAP	GSK3A	DCTN4
HTATSF1	TAF13	GTPBP4	DCTN6
ID3	TRP53	H1F0	DDOST
IK	TUBB6	HDGF	DEGS1
ILF3	VDAC3	HIC1	DHRS1
IVNS1ABP	VIM	HIPK2	DNAJA1
KANSL1	YBX1	HMGN1	DNAJB11
KHDRBS1	YEATS4	HNRNPA3	DPP8
KLF3	ZFP207	HNRNPH2	EGLN1
KPNB1		HNRNPLL	EIF4EBP2
LEO1		HNRNPUL1	EIF4ENIF1
LRRC59		ILK	ELK4
LSM14A		KLHDC2	ELOVL5
MBD2		KLHL9	EMC3
MFSD1		LARP4B	EMC4
MSL1		LASP1	EMC7
MYH9		LIX1L	EP300
NCBP2		LSM12	ERGIC3
NDUFA12		MAP4	ERP29
NEDD4		MAP7D1	ESYT1
NUP62		MAPK1IP1L	ETFA
P4HB		MAPKAP1	FADS1
PABPC1		MAPRE1	FAM114A1
PAICS		MAPRE2	FBXO22
PCBP1		MIDN	GANAB
PCBP2		MRFAP1	GM13363
PFN1		MTDH	GM6644
PNRC2		MTSS1L	GOLGA4
POLR2A		NDUFS2	GORASP2
PPP1CC		NONO	GRN
PPP1R12A		NRBP1	GTF2B
PPP3R1		NUCKS1	H2-K1
PRELID1		PABPN1	HADHA
PRKAR1A		PAPOLA	HADHB
PRPF40A		PCM1	HAX1
PRPF4B		PDCD5	HDLBP
PRRC2A		PDS5A	HECTD1
PSMD11		PICALM	HIAT1
PSMD12		PITPNA	HIPK1
PSMD6		POLR2M	HNRNPUL2
PTBP1		PPP1CA	HSP90B1
PTP4A2		PSMA7	IFITM2
PTPN12		PSMC2	IFT20
QK		PSMD1	IQGAP1
RAB10		PTBP3	IRF2BP2
RAB14		PTCH1	ITFG1
RAB6A		PTOV1	JAGN1
RAC1		PUM2	KCMF1
RNF187		RAD21	KDELR2
RNF7		RAP1A	KIF1B
SCAF11		RBBP6	KLF6
SENP6		RERE	KMT2E
SH3BGRL3		REST	KRCC1
SH3GLB1		RRP1	LAMP1
SLTM		SETD8	LAMTOR5
SMC6		SHFM1	LGALS9
SRP72		SHOC2	LIMA1
SRRM1		SKAP2	LIMS1
SRSF2		SKIV2L2	LMAN1
SRSF3		SLK	MAPK3
SRSF5		SMARCA4	MAT2A
SSR3		SMARCE1	MAX
STAG1		SMO	MEF2A
STMN1		SNAI2	MLF2
STX12		SNHG5	MYL12A
SUPT16		SNX4	NBR1
TAB2		SP1	NCOA4
TBL1X		SPIN1	NCSTN
TCF12		SUCLG2	NDFIP1
TFG		SYNCRIP	NFE2L1
THRAP3		TCEA1	NFIX
TMED9		THOC7	NISCH
TMEM123		TOMM22	OCIAD1
TMEM131		TPR	PAFAH1B2
TMEM234		TSPAN3	PDHB
TMPO		UBE2E3	PDIA4
TOP2B		UBE2I	PDZD11
TRIP12		UBE2V1	PHLDA1
TTC3		UBE4B	PLXNB2
TUBA1A		USP47	PPP2R1A
TUBB5		UTP3	PPT1
VAMP3		VCP	PSMC5
VDAC2		WAPAL	PTPRS
WDR1		WDR26	RAB1
XRN2		YWHAB	RAB7
YTHDC1		ZC3H15	RAB9
YWHAE		ZFP664	RANBP9
YWHAH		ZMYND11	RAP2A
YWHAQ			RCN1
ZCRB1			REEP5
			RHOB
			RNH1
			RTN3
			S100A11
			SAR1A
			SAR1B
			SEC. 31A
			SEC. 61A1
			SEC. 63
			SERINC1
			SIRT2
			SMDT1
			SNAPIN
			SRPR
			STAU1
			STT3A
			STT3B
			STX4A
			STX5A
			SUPT6
			SWI5
			TAGLN2
			TAOK1
			TCF25
			TECR
			TLN1
			TM9SF2
			TMBIM6
			TMED10
			TMED2
			TMOD3
			TOR1AIP2
			TRAPPC6B
			TRP53BP2
			TUBB4B
			TXNDC5
			UBR5
			UBXN4
			UGP2
			USP16
			USP8
			VGLL4
			VPS25
			VPS28
			YWHAG
			ZFP106
			ZMIZ1
			ZYX

## 2 Experimental design, materials and methods

### 2.1 Animals

B6.129S4-*PDGFRα*^*tm11**(EGFP)**Sor*^/J mouse-line herein designated PDGFRα^GFP^ [2], with PDGFRα promoter driving the expression of the H2B-eGFP fusion gene were used for immunohistochemical, differential plate-down, and flow cytometry analyses. Mice lacking the PDGFRα GFP tag were used for PDGFRα^+^ cell RNA-Seq analysis.

### 2.2 Confocal microscopy

Lung tissues were harvested, fixed with 4% PFA in PBS and frozen. Tissue was sectioned into 200 μm slices and stained with anti-αSMA (Sigma-Aldrich, St. Louis, MO), Pro-SPC and chicken polyclonal anti-GFP antibody (Abcam, Cambridge, MA). Data was analyzed by Imaris software, version 7.6.

### 2.3 Characterization of PDGFRα^GFP^ Cells by flow cytometry in plate-adhered or suspension cells

Lung tissue from PDGFRα^GFP^ mice was harvested, processed into single cell suspension as previously described [3]. Cells were incubated in Dulbecco׳s DMEM/F12 (10% FBS, 2% pen/strep) after 2 h of culture, the media containing the non-adherent cell fraction was collected, and the adherent fraction was collected using Accutase (1× ACCUTASE enzymes in Dulbecco׳s PBS (0.2 g/L KCl, 0.2 g/L KH_2_PO_4_, 8 g/L NaCl, and 1.15 g/L Na_2_HPO_4_) containing 0.5 mM EDTA·4Na and 3 mg/L Phenol Red).

### 2.4 Bioinformatics data analysis

RNA-Seq data was quantitated using TopHat and Cufflinks [4], genes were included with the expression level (FPKM) was more than 1 in all samples. Bayesian Analysis of Time Series (BATS) identified genes as differentially expressed at one or more timepoints, co-regulated genes were identified by using pattern recognition using STEM and grouped into Gene expression profiles. Gene expression profiles were subjected to gene set enrichment analysis with Toppgene and Toppcluster [5], [6], [7].

### 2.5 Transcription factor promoter enrichment analysis

Transcription factor promoter enrichment analysis of PDGFRα^+^ fibroblast RNA-Seq profiles identified three candidate transcription factors: NRSF/REST, CTCF, and MAX. ChIP-Seq has been performed in the following mouse cell lines, and the data available in the public domain:

MAX: http://www.ncbi.nlm.nih.gov/geo/query/acc.cgi?acc=GSM912908
CTCF: http://www.ncbi.nlm.nih.gov/geo/query/acc.cgi?acc=GSM918744
NRSF/REST: https://www.encodeproject.org/experiments/ENCSR000AIS/

To identify potential candidate genes in PDGFRα^+^ fibroblasts regulated by NRSF/REST, CTCF, or MAX during acinar lung development, we cross-referenced the dynamically regulated genes identified by our present RNA-Seq analysis with the above, previously-published gene sets [1].
